# AKR1B10 confers resistance to radiotherapy via FFA/TLR4/NF-κB axis in nasopharyngeal carcinoma

**DOI:** 10.7150/ijbs.52927

**Published:** 2021-02-03

**Authors:** Xiangting Liu, Zheng Hu, Jiayao Qu, Jia Li, Ke Gong, Li Wang, Jing Jiang, Xiangning Li, Rongzhang He, Lili Duan, Weihao Luo, Chenglai Xia, Dixian Luo

**Affiliations:** 1 Translational Medicine Institute, the First People's Hospital of Chenzhou, University of South China, Hunan 432000, P.R China.; 2 The First School of Clinical Medicine, Southern Medical University, Guangdong Guangzhou 51000, China.; 3 South Medical University Affiliated Maternal & child Health Hospital of Foshan, Foshan 528000, P.R. China.; 4 School of Pharmaceutical Sciences, Southern Medical University, Guangzhou 520150, P.R. China.; 5 Department of Laboratory Medicine, Huazhong University of Science and Technology Union Shenzhen Hospital (Nanshan Hospital), Guangdong 518000, P.R China.; 6 Center for Laboratory and Pathology, National & Local Joint Engineering Laboratory for High-through Molecular Diagnosis Technology, the First People's Hospital of Chenzhou, Southern Medical University, Hunan 423000, P.R China.

**Keywords:** nasopharyngeal carcinoma, AKR1B10, radiotherapy resistance, FFA/TLR4/NF-κB axis

## Abstract

Nasopharyngeal carcinoma (NPC) is one kind of human head and neck cancers with high incidence in Southern China, Southeast Asia and North Africa. In spite of great innovations in radiation and chemotherapy treatments, the 5-year survival rate is not satisfactory. One of the main reasons is resistance to radiotherapy which leads to therapy failure and recurrence of NPC. The mechanism underlying remains to be fully elucidated. Aldo-keto reductase B10 (AKR1B10) plays a role in the formation and development of carcinomas. However, its role in resistance to radiotherapy of NPC is not clear. In this research, the relationships between AKR1B10 expression and the treatment effect of NPC patients, NPC cell survival, cell apoptosis, and DNA damage repair, as well as the effect and mechanism of AKR1B10 expression on NPC radioresistance were explored. A total of 58 paraffin tissues of NPC patients received radiotherapy were collected including 30 patients with radiosensitivity and 28 patients with radioresistance. The relationships between AKR1B10 expression and the treatment effect as well as clinical characteristics were analyzed by immuno-histochemical experiments, and the roles of AKR1B10 in cell survival, apoptosis and DNA damage repair were detected using the AKR1B10 overexpressed cell models. Furthermore the mechanism of AKR1B10 in NPC radioresistance was explored. Finally, the radioresistance effect of AKR1B10 expression was evaluated by the tumor xenograft model of nude mice and the method of radiotherapy. The results showed AKR1B10 expression level was correlated with radiotherapy resistance, and AKR1B10 overexpression promoted proliferation of NPC cells, reduced apoptosis and decreased cellular DNA damage after radiotherapy. The probable molecular mechanism is that AKR1B10 expression activated FFA/TLR4/NF-κB axis in NPC cells. This was validated by using the TLR4 inhibitor TAK242 to treat NPC cells with AKR1B10 expression, which reduced the phosphorylation of NF-κB. This study suggests that AKR1B10 can induce radiotherapy resistance and promote cell survival via FFA/TLR4/NF-κB axis in NPC, which may provide a novel target to fight against radiotherapy resistance of NPC.

## Introduction

Nasopharyngeal carcinoma (NPC) is a highly metastatic and aggressive malignant tumor prevalent in southern China 1. Ionizing radiation (IR) is the primary therapeutic option for NPC. However, resistance to radiotherapy leads to therapy failure and recurrence of NPC, thus reducing the quality of life of NPC patients. It is therefore important to elucidate the mechanism underlying the radiation resistance in NPC patients.

IR directly induces DNA damage, largely by introducing single or double-stranded breaks in the DNA. Irreversible DNA damage leads to cell senescence, mitotic disorders, necrosis and/or induction of apoptosis 2. To maintain genomic integrity, the DNA damage response (DDR) is rapidly activated. Ataxia telangiectasia mutated (ATM) activation and phosphorylation is a main event occurring during the DDR. ATM-mediated signaling contributes to cell cycle arrest and downstream DNA repair. Subsequently, a histone variant H2AX is phosphorylated at serine 139 (γH2AX) when double-stranded breaks (DSBs) occur in cells. γH2AX is a biomarker of the cellular response to DSBs and can reflect the status of DNA damage and repair. Compared to normal cells, tumor cells are actively dividing, and DNA damage repair mechanisms are often defective and DNA damage cannot be repaired. In order to deal with DDR, the RAD9-HUS1-RAD1(9-1-1) complex is recruited to the site of DNA damage and promotes ATR-mediated phosphorylation and activation of CHK1, a human effector checkpoint protein kinase which regulates S phase progression and G2/M cell cycle arrest^3^. The MRE11-RAD50-NBS1 (MRN) complex identifies DSBs, which then recruits ATM and promotes the formation of histone-intervened H2AX (γH2AX) 4,5. This endogenous resistance to radiotherapy leads to local recurrence and distant metastasis of cancer.

Aldo-Keto Reductase Family 1 B10, AKR1B10, is a member of the aldo-ketoreductase superfamily. AKR1B10 may play a role in cancer development and progression through a variety of molecular mechanisms including the detoxification of cytotoxic carbonyls, modulation of retinoic acid levels, and regulating cellular fatty acid synthesis and lipid metabolism 6-8. So we suggested that AKR1B10 may protect cells from DNA damage during radiotherapy and thus may play an important role in resistance to radiotherapy of NPC.

Nuclear factor-kappa B (NF-κB) signaling is involved in radiotherapy resistance of NPC, and inhibition of NF-κB enhances sensitivity to radiotherapy 9. NF-κB is a group of nuclear protein factors that regulate the expression of a wide array of genes involved in the regulation of cellular physiological processes, immune response and tumorigenesis. Inhibitor of κB (IκB) regulates NF-κB activity 10. When unstimulated, NF-κB is sequestered in the cytoplasm through interactions with IκBs 11, which cover the NF-κB nuclear localization and prevents nuclear translocation. When DNA damage occurs, the damage signal leads to IκB phosphorylation and subsequent ubiquitination and degradation. This exposes the nuclear localization sequence of NF-κB to induce the nuclear translocation of NF-κB, thereby initiating expression of the target genes 12.

Toll like receptor 4 (TLR4) is a key pro-inflammatory factor. Due to the unique physiological and anatomical sites of NPC, nasopharyngeal epithelial cells are susceptible to EB virus and other pathogens, which develop into chronic inflammation ultimately aiding in cancer development 13. NF-κB is an important transcription factor downstream of TLR4. IκB phosphorylation and the subsequent NF-κB nuclear translocation is characteristic of TLR4 activation 14. Ma et al found that AKR1B10 can increase the synthesis of FFA by stabilizing a rate-limiting enzyme of lipid synthesis, ACCα, after association with it, which protects ACCα avoiding degradation through the ubiquitination-proteasome pathway 15. Another report found that the TLR4/NF-κB signaling could be activated after treated with FFA in adipocytes and macrophages 16. This study demonstrated that AKR1B10 activates FFA/TLR4/NF-κB axis to induce radiotherapy resistance of NPC in some cases.

## Materials and Methods

### Patients and tissues

This study was approved by the Ethics Review Committee of the First People's Hospital of Chenzhou. 58 NPC patients received radiotherapy during January 2016 to December 2018 were selected in this study. According to standardized evaluation criteria of published literature, the NPC patients were defined as two groups, the radioresistant and the radiosensitive 17-20. The expression level of AKR1B10 was detected by immunohistochemistry (IHC) staining. The pathological diagnosis of all patients' specimens was confirmed by two experienced pathologists 21.

### Cell culture and irradiations

NPC cell line CNE-2, and its derived cells, CNE-2/Vector and CNE-2/AKR1B10, were cultured in suitable conditions 22. Cells were cultured in a multi-well plate for 6-MV X-ray treatment and the cells were harvested at corresponding time points.

For irradiation, the cells or the tumors were irradiated at defined doses using a Siemens PRIMUS X-ray irradiator (2 Gy/min, 6-MV, 15cm×10cm, SAD 100cm, d=5.5cm; Rad Source Tech, PRIMUS ,Siemens, Germany). In our study, the radiation doses of 2, 4 and 6 Gy with a dose rate of 2 Gy/min were used, thus the duration of IR treatment are 1min, 2 min or 3 min respectively.

### Lentiviral vector construction, virus infection and the establishment of stable cell line

The AKR1B10 CDS (coding DNA sequence) was cloned into the lentiviral expression vector pSIN-EF2-Puro (hereinafter referred to as pSIN-EF2) to construct the lentiviral vector pSIN-EF2/AKR1B10. The method to establish stable AKR1B10-expression cell line has been described previously 23.

### AKR1B10-shRNA stable transfection using lentiviral infection

The PLKO.1-puro vector was used to clone the sh-RNAs targeting AKR1B10. The sequences of shAKR1B10 were as followed. shRNA1: 5′-CCGGAAGTGAAAGAAGCAGT GAAGGCTCGAGCCTTCACTGCTTCTTTCACTTTTTTTG-3′; shRNA2: 5′-CCGGTGTT GCAATCCTCTCATTTGGCTCGAGCCAAATGAGAGGATTGCAACATTTTTG-3′. The plasmids were transfected into HEK293T cells and the supernatant which contained the virus was collected at 48 and 72 h. The virus was then concentrated and transfected in CNE-2/AKR1B10 cells with polybrene (Sigma-Aldrich Co.,USA). The transfected cells were selected by puromycin for at least 1 week.

### Quantitative real-time PCR

Quantitative real-time PCR (qRT-PCR) was performed as previous report 24. Briefly, total RNA was isolated using Trizol® reagent (Molecular Research Center, USA) and 2 μg RNA was subjected to reverse transcription reaction to obtain cDNAs (Reverse Transcription System, promega, USA). Specific quantitative real-time PCR experiments were performed using SYBR Green Power Master Mix following the manufacturer's protocol (Applied Biosystems). Glyceraldehyde-3-phosphate dehydrogenase (GAPDH) was used as the internal control. The following primers were used: AKR1B10 forward (5'-CCCAGGAGACAGAGGTTATA-3') and reverse (5'-GAAATGATTCTGAGTGAGCAGGTAG-3'); GAPDH forward (5'-ACCACAGTCCATGCCATCAC-3') and reverse (5'-TCCACCCTGTTGCTGTA-3').

### Clonogenic assays

Cells were seeded in cell culture dish and irradiated with various doses of γ-radiation at room temperature. The cells were cultured in an incubator for 12 to 14 days. Cells were fixed and stained with a solution containing 0.5% crystal violet in methanol and the dishes with colonies of >50 cells were counted manually.

### Annexin V-APC/7-AAD Apoptosis Assay

Cells were seeded in a cell culture dish, after culture cells for 24 h, exposed to radiotherapy. After 48 h, the cells were harvested and stained with the Annexin V-APC/7-AAD Apoptosis Detection Kit (BestBio, BB-41034, China). The stained samples were analyzed by flow cytometry (MoFlo XDP, Beckman, CA, USA).

### Immunofluorescence staining

Cells were plated into each well of 6-well plates. After 24 h, the cells were exposed to 2 Gy radiation. The cells were fixed with 4% paraformaldehyde (Sigma) for 15 min. The cells were incubated with primary antibody, i.e., mouse anti-phospho-Histone H2AX (Ser139) (provide source) and subsequent incubation with Dylight 488, Goat Anti-Rat IgG secondary antibody. The cells were counter-stained with DAPI. Images were acquired.

### Neutral comet assay

Cells were cultured in 6-well plates. After 24 h, the cells were irradiated with 6Gy and were collected for the experiment. The length of the appendix was calculated using the CASP software. Tail moment was used as the parameter of DNA damage using the following formula: Tail moment = fraction of DNA in the tail × tail length.

### FFA ELISA assay

Total 5×10^6^ cells were collected and used for FFA assay. After centrifuge, the cell pellet was resuspended with 1×PBS to make the cell concentration reach to 1 million/ml. Then the cells were broken by ultrasound and the supernatant collected after centrifuge, and this was used for ELISA assay. FFA concentation was quantified with the human free fatty acid ELISA kits (Jianglai Bio, China) to detect cell FFA in cell lysates.

### FFA and TLR4 inhibitor treatment

Cells were cultured in 6-well plates. The cells treated with FFA at 50 μM, and continue to be cultivated for 24 h, then the cells were treated with TLR4 inhibitor (TLR4-IN-C34) at a concentration of 10 μM, 50 μM and 100 μM in CNE-2/AKR1B10 cells. After 48 hours, the cells of each group including the control were collected and the protein was extracted for western blot analysis.

### Tumorigenesis assay in tumor xenograft model of nude mice

5-week-old female BALB/C nude mice were purchased from the Hunan SJA Laboratory Animal Company (Changsha, China) and were maintained under specific pathogen-free conditions. 24 mice were used for the experiment and four groups were established randomly. Six mice in each group were subcutaneously injected with 2×10^6^ cells (CNE-2/Vector cells or CNE2/AKR1B10 cells) into the right flank. Tumor volume was monitored every two days and when the tumor volumes reached 100-200 mm^3^, the tumors were exposed to 6 Gy IR. Two weeks after IR, tumor volume was measured and calculated. The study protocol complied with the ARRIVE guidelines and was carried out by following the National Institutes of Health guide for the care and use of Laboratory animals.

### Western blotting

Total protein was extracted by using RIPA buffer, quantified by BCA assay, and subjected to SDS-PAGE gel electrophoresis and transferred to nitrocellulose membrane (NC). The blot was blocked with 5% BSA or 5% non-fat milk, and then incubated respectively wi**t**h anti-AKR1B10 antibody (prepared by our laboratory), p-chk1/2 (Catalog #AC506, #AC508, Beyotime), chk1/2 (Catalog # 2662, #2360, CST), cleaved-PARP (Catalog #5625, CST), PARP (Catalog #9524, CST), cleaved-caspase3 (Catalog #9664, CST), caspase3, p-NFκB (Thr183/Tyr185) (Catalog #4671, CST), NF-ΚB (D14E12) (Catalog #8242,CST), p-IKBα (Ser473) (Catalog #4060, CST), IKBα, TLR4 (Catalog #35463,SAB), MyD88 (Catalog #41191, SAB), β-actin (Catalog #60008-1, Proteintech) overnight at 4 °C. Then the blot was incubated with the Goat anti-rabbit secondary antibody Catalog #ZB-2301, ZSGB-BIO) or Goat anti-Mouse secondary antibody (Catalog #ZB-2305, ZSGB-BIO) for 1.5 hours on a room temperature. After extensive washing in PBST, the expression levels of the proteins were detected by Quantity-one software (Bio-Rad Laboratories, USA) using the ECL-chemiluminescent kit. The signal of protein bands was quantified by ImageJ software for Windows (NIH, USA).

### Statistical analysis

All the cell experiments were repeated three times. The differences between the control group and the experimental groups were determined by Student's t test. Data was analyzed using SPSS 19 statistical software (SPSS, Inc, Chicago, IL, U.S.A.), and the results were considered significant when the p value <0.05.

## Results

### AKR1B10 is associated with the radioresistance of NPC

**Figure 1:**
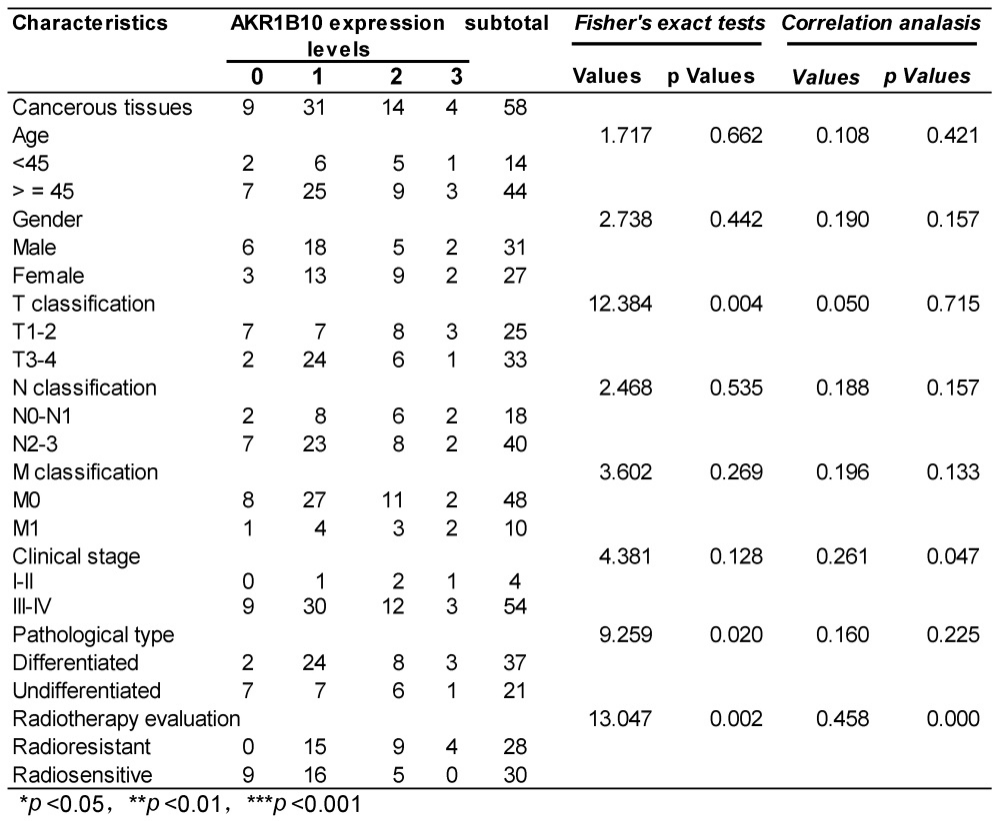

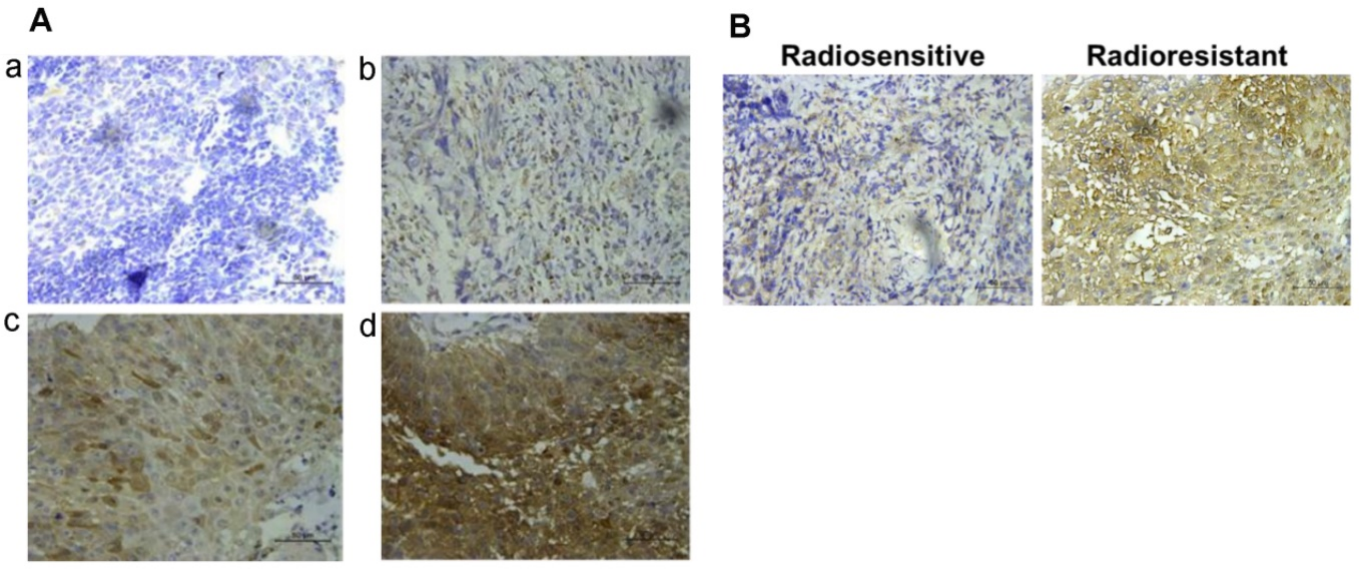
** The expression of AKR1B10 in patients of radiotherapy resistance and radiotherapy sensitivity.** (A) AKR1B10 expression detected by immunochemistry, and images a-d are representative images of 4 levels of expression from 0 to 3 score, such as 0 for no staining (a), 1 for week staining (b), 2 for moderate staining (c), 3 for strong staining (d). (B) Immunohistochemical detection of AKR1B10 expression in radio-sensitive and radio-resistant patients.

58 NPC patients accepting radiotherapy were recruited in this study. According to the literature evaluation criteria, they were divided into 28 radioresistance patients and 30 radiosensitive patients. AKR1B10 expression levels were detected in the NPC tissues obtained before any anti-cancer therapy. Immunohistochemical score analysis of AKR1B10 expression revealed a range of expression levels in NPC (representative images shown in Fig. 1a), and higher expression of AKR1B10 detected in radiation resistant patients (Fig. 1b). Clinical and histopathological data of the NPC patients are presented in Table 1. AKR1B10 expression levels in the radioresistant group was significantly higher and stronger than that in radiosensitive group (P=0.002). There were no significant differences in age (P=0.662), gender ratio (P=0.442), N classification (P=0.535), M classification (P=0.269) and clinical stage (P=0.126).

### AKR1B10 enhances the radioresistance of NPC cells

**Figure 2:**
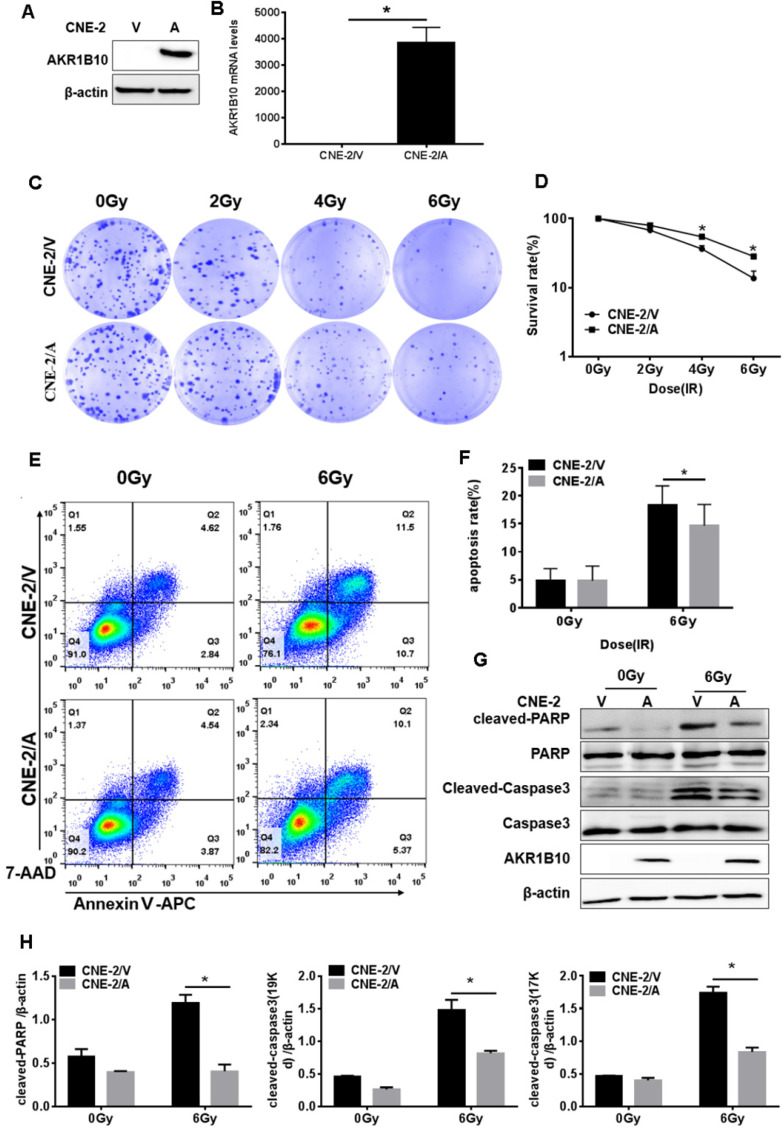
** AKR1B10 forcing expression promoted proliferation and reduced apoptosis of NPC cells**. (A and B) AKR1B10 expression confirmed at CNE-2 cells by Western blot and qRT-PCR. (C and D) The proliferation of CNE-2/AKR1B10 cells verified by clone formation assay. The experiments repeated three times. (E and F) The effect of AKR1B10 expression on CNE-2 cell apoptosis detected by Flow cytometry after IR treatment. (G) Expression levels of cleaved-caspase-3, cleaved-PARP in CNE-2/AKR1B10 cells after IR (6 Gy) 48 hours determinated by Western blot. (H) Quantification and statistics for the cleaved PARP and cleaved Caspase 3 expression. IR: irradiation; Gy: GrayA; AKR1B10; V: vector, psin-EF2-puromycine; CNE-2/A: CNE-2/AKR1B10, AKR1B10 expressed CNE-2 cells; CNE-2/V: CNE-2/AKR1B10 vector, CNE-2 control cells; *p< 0.05.

CNE-2 cells with stable overexpression of AKR1B10 were constructed. AKR1B10 protein and mRNA levels were detected in the CNE-2/AKR1B10 by western blot and real-time fluorescent quantitative PCR (RT-PCR) (Fig. 2a and b). We performed colony formation assays and calculated the survival rate of the cells. As showed in Fig. 2c and 2d, AKR1B10 overexpression significantly increased the survival rate of CNE-2 cells treated with 4 or 6 Gy radiation. We checked the change in cell apoptosis by flow cytometry using Annexin V-APC/7-AAD staining. AKR1B10 overexpression significantly decreased the rate of apoptosis in CNE-2 cells treated with 6 Gy radiation (Fig. 2e and f). We also detected the activation of caspase-3 and poly (ADP-ribose) polymerase (PARP) (Fig. 2g and 2h), which are the markers of cell apoptosis. Cleaved PARP and cleaved caspase-3 significantly decreased in AKR1B10 overexpression cells after IR treatment for 48h. These results suggest that AKR1B10 overexpression enhances cell radiotherapy resistance, increases cell survival, and reduces cell apoptosis.

### AKR1B10 reduces DNA damage and cell cycle arrest in NPC cells

**Figure 3:**
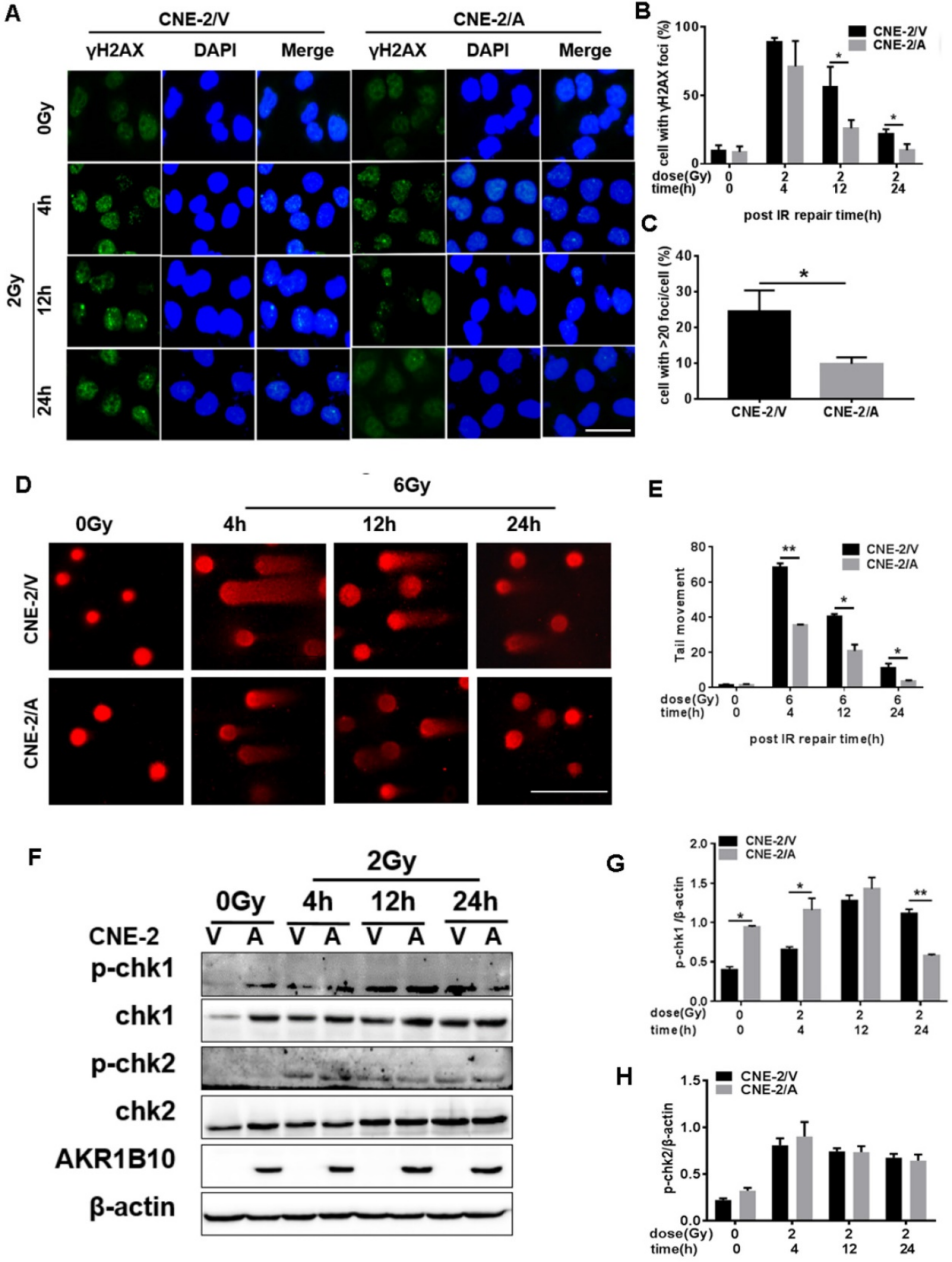
** Evaluation of DNA damage after irradiation in AKR1B10-overexpression NPC cells.** (A) The γH2AX staining of CNE-2/AKR1B10 cells (the scale bar represents 50 µm). (B and C) Statistics of the percentage of γH2AX foci and foci greater than 20 in CNE-2/AKR1B10 and CNE-2/vector cells. (D and E) The comet assay of CNE-2/AKR1B10 cells after IR treatment. D is one of the representative Images and E is the statistics result of three expreiments (counting 100 cells per group, Scale bar 200μm). (F) The effect of AKRB10 expression on cell cycle checkpoint proteins expression by Western blot after IR treatment. (G and H) The relative quantification analysis of the change of phosphorylated CHK1 and CHK2 between the control group and the experimental groups. The signals of protein bands relative to β-actin in three experiments were quantified by ImageJ software. IR: irradiation; Gy: GrayA; A: AKR1B10; V: vector, psin-EF2-puromycine; CNE-2/A: CNE-2/AKR1B10, AKR1B10-expressed CNE-2 cells; CNE-2/V: CNE-2/AKR1B10 vector, CNE-2 control cells; p-CHK1: phosphorylated CHK1; p-CHK2: phosphorylated CHK2. *P < 0.05; **P < 0.01.

To evaluate IR-induced DNA damage during radiotherapy, we evaluated the level of γH2AX as an indicator of double-strand breaks (DSB) after IR by immunofluorescence assay. AKR1B10 overexpression significantly decreased the number of γ-H2AX foci in the CNE-2 cell lines at both 12 h and 24 h after 2 Gy radiation (Fig. 3a and b). We also calculated the number of the cells with more than 20 γH2AX foci (Fig. 3c), and arrived at the similar conclusion that AKR1B10 overexpression alleviated DNA damage. At the same time, we performed single-cell gel electrophoresis (neutral comet assay) and measured tail moment (tail moment = fraction of DNA in the tail×tail length), which positively correlated with the level of DNA damage in a cell 25. Tail moment was shorter in CNE-2/AKR1B10 than CNE-2/Vector, suggesting that AKR1B10 overexpression inhibits DNA damage undergoing repair (Fig. 3d and e).

CHK1 and CHK2, two important effectors of cell cycle progression, are phosphorylated when DNA damage happens, which leads to cell arrest, thus giving the cell time to repair DNA 11. We found that AKR1B10 overexpression induced the expression levels of CHK1 (Fig. 3f & 3g). The phosphorylation level of CHK1 was higher in CNE-2/AKR1B10 at 4h after IR treatment than CNE-2/Vector. However, at 24 h after IR, the phosphorylation level of CHK1 was significantly lower in CNE-2/AKR1B10 than CNE-2/Vector, suggesting that AKR1B10 may induce the cells to repair DNA damage. However the AKR1B10 expression did not affect the expression of CHK2 or its phosphorylation induced by IR (Fig. 3f & 3h).

### AKR1B10 promotes NPC radioresistance through activation of the NF-κB pathway

**Figure 4:**
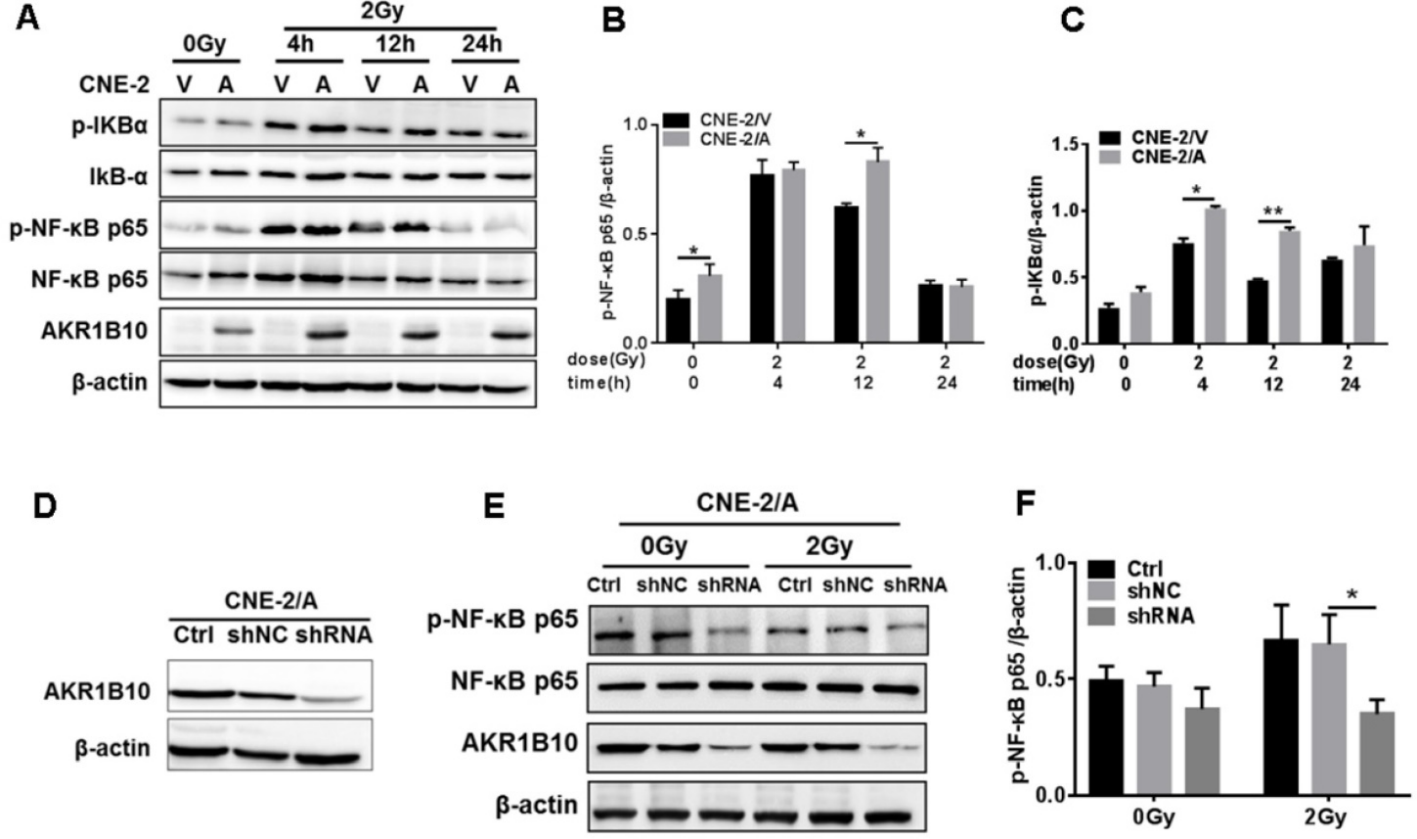
** AKR1B10 activates the NF-κB pathway.** (A) The levels of IKBα/NF-κB signaling pathway protein at CNE-2/AKR1B10 cells were assessed by Western blot after IR treatment (0 and 2 Gy) at 4, 12 and 24 h respectively. (B and C) The quantificated statistics results of phosphorylated NF-κB and phosphorylated IKBα protein expression relative to β-actin protein level. The experiments were repeated three times. (D) Construction of AKR1B10-knockdown cell line. (E and F) Knockdown of AKR1B10 downregulates the phosphorylation level of NF-κB. IR: irradiation; Gy: GrayA; A: AKR1B10; V: vector, psin-EF2-puromycine; CNE-2/A: CNE-2/AKR1B10, AKR1B10 expressed CNE-2 cells; CNE-2/V: CNE-2/AKR1B10 vector, CNE-2 control cells; *P < 0.05; **P < 0.01.

Activation of the NF-κB signaling pathway plays an important role in the radioresistance of tumor cells 26. Inactivated NF-κB always binds to IκB to become homodimer, and when NF-κB is activated by phosphorylation, IκB will be phosphorylated to be degraded 27. We found that IR induced the expression of phosphorylated NF-κB and IKBα at 4 h and 12 h, followed by a decline in their expression and phosphorylation levels. AKR1B10 overexpression enhances the phosphorylation levels of NF-κB and IKBα at 4 h and 12 h after IR (Fig. 4a, b and c). Furthermore, we used a lentivirus system to construct an AKR1B10 knockdown cell line (Fig. 4d). AKR1B10 silencing also inhibited the phosphorylation of NF-κB 8h after IR (Fig. 4e and f). These studies show that AKR1B10 can increase NF-κB activity, which suggested that AKR1B10 contributed to NPC radioresistance by activating the IKBα/NF-κB signaling pathway.

### AKR1B10 activates TLR4/NF-κB signaling pathway by increasing FFA in NPC

**Figure 5:**
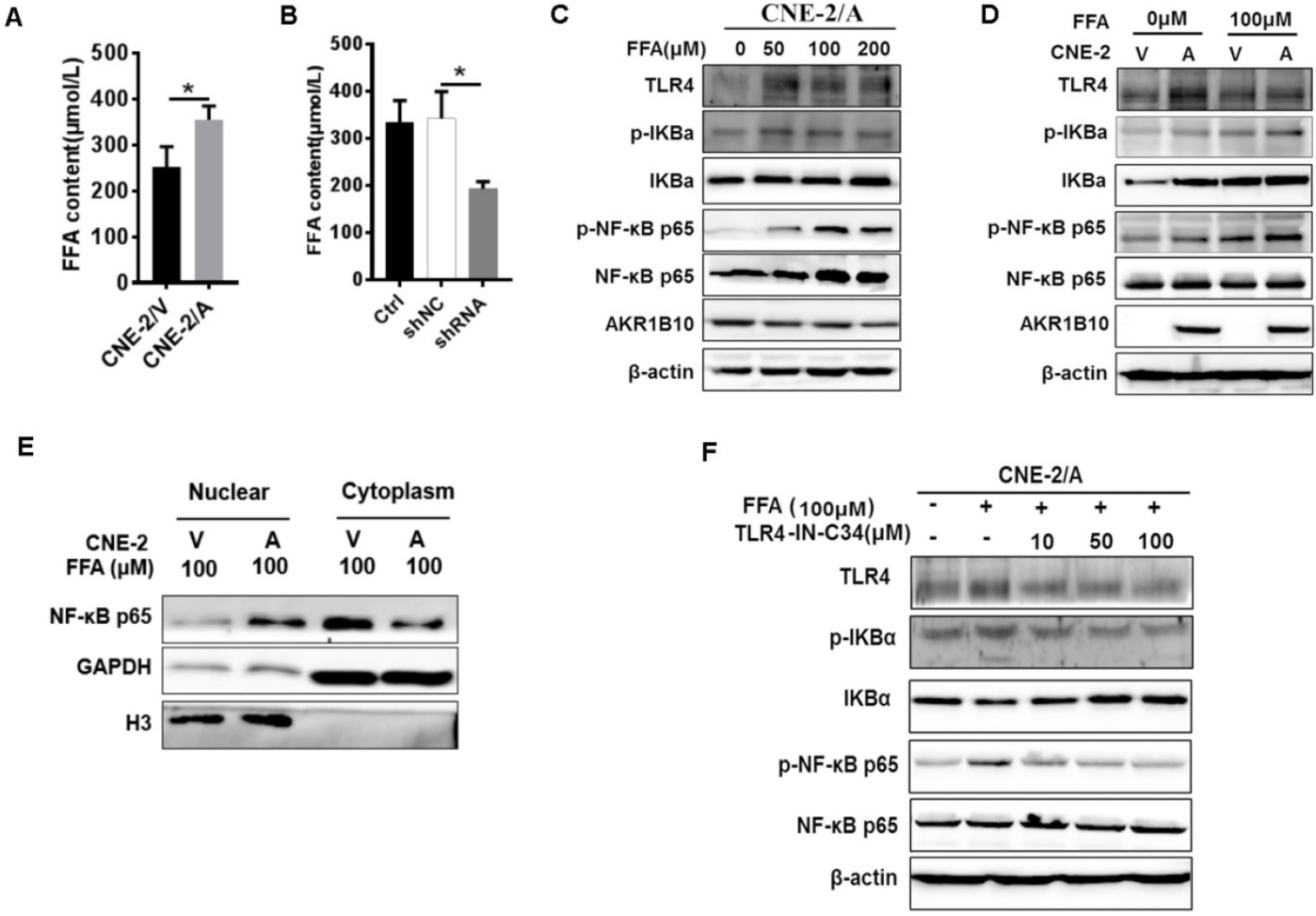
** Enhanced AKR1B10 expression activates the TLR4/ NF-κB signaling pathway.** (A and B) The effect of AKR1B10 expression on FFA content. AKR1B10 expression increases FFA content of CNE-2/AKR1B10 cells (CNE-2/A), meanwhile, knockdown of AKR1B10 expression decreases FFA content. (C) 0, 50, 100 and 200 µM FFA were used to treat CNE-2/AKR1B10 cells and the expression levels of TLR4, p-IKBɑ and p-NF-κB p65 as well as AKR1B10 were detected by western blot. (D) The expression levels of TLR4, p-IKBɑ and p-NF-κB p65 as well as AKR1B10 were further confirmed in the CNE-2/vector and CN-2/AKR1B10 cells after treated with 100 μM FFA. (E) The effect of FFA on NF-κB p65 entry into the nucleus. (F) The effect of FFA inhibitors on TLR4 signaling pathway. A: AKR1B10; V: vector, psin-EF2-puromycin; CNE-2/A: CNE-2/AKR1B10, AKR1B10 expressed CNE-2 cells; CNE-2/V: CNE-2/AKR1B10 vector, CNE-2 control cells; FFA: Free fatty acid; H3: H3 histone; TLR4-IN-C34:TLR4 inhibitor; p-IKBɑ: phosphorylated IKBα; p-NF-κB: phosphorylated NF-κB. *P < 0.05.

AKR1B10 promotes FFA synthesis by stabling ACCα 15. To elucidate the precise mechanisms by which AKR1B10 induces radioresistance of NPC cells, we detected the FFA content in the NPC cells. The FFA level was significantly increased in CNE-2/AKR1B10 (Fig. 5A), and significantly decreased in CNE-2/AKR1B10 with AKR1B10 shRNA transfection (Fig. 5B). Then we used 0, 50, 100 and 200 μM FFA to treat CNE-2/AKR1B10 to detect whether the NF-κB signaling was activated. The results showed that the level of phosphorylated NF-κB (p-NF-κB) was increased with the FFA concentration increasing. The expression level of TLR4 and phosphorylated IKBα (p-IKBα) also increased in FFA treated cells (Fig. 5C). This result was further confirmed in the CNE-2/vector and CN-2/AKR1B10 cells after treated with 100 μM FFA (Fig. 5D). Moreover, exogenous FFA significantly induced nuclear translocation of NF-κB P65 (Fig. 5E). Furthermore, an inhibitor of TLR4 signaling, TLR4-IN-C34, which is an aminomonosaccharide that inhibits TLR4 signaling by docking with the hydrophobic pocket of the TLR4 co-receptor, myeloid differentiation protein-2 (MD-2), could block FFA-induced NF-κB activation in CNE-2/AKR1B10 (Fig. 5F). These results illustrated that AKR1B10 could activate TLR4/NF-κB signaling pathway via promoting the FFA synthesis in NPC.

### AKR1B10 promotes radioresistance of NPC *in vivo*

**Figure 6:**
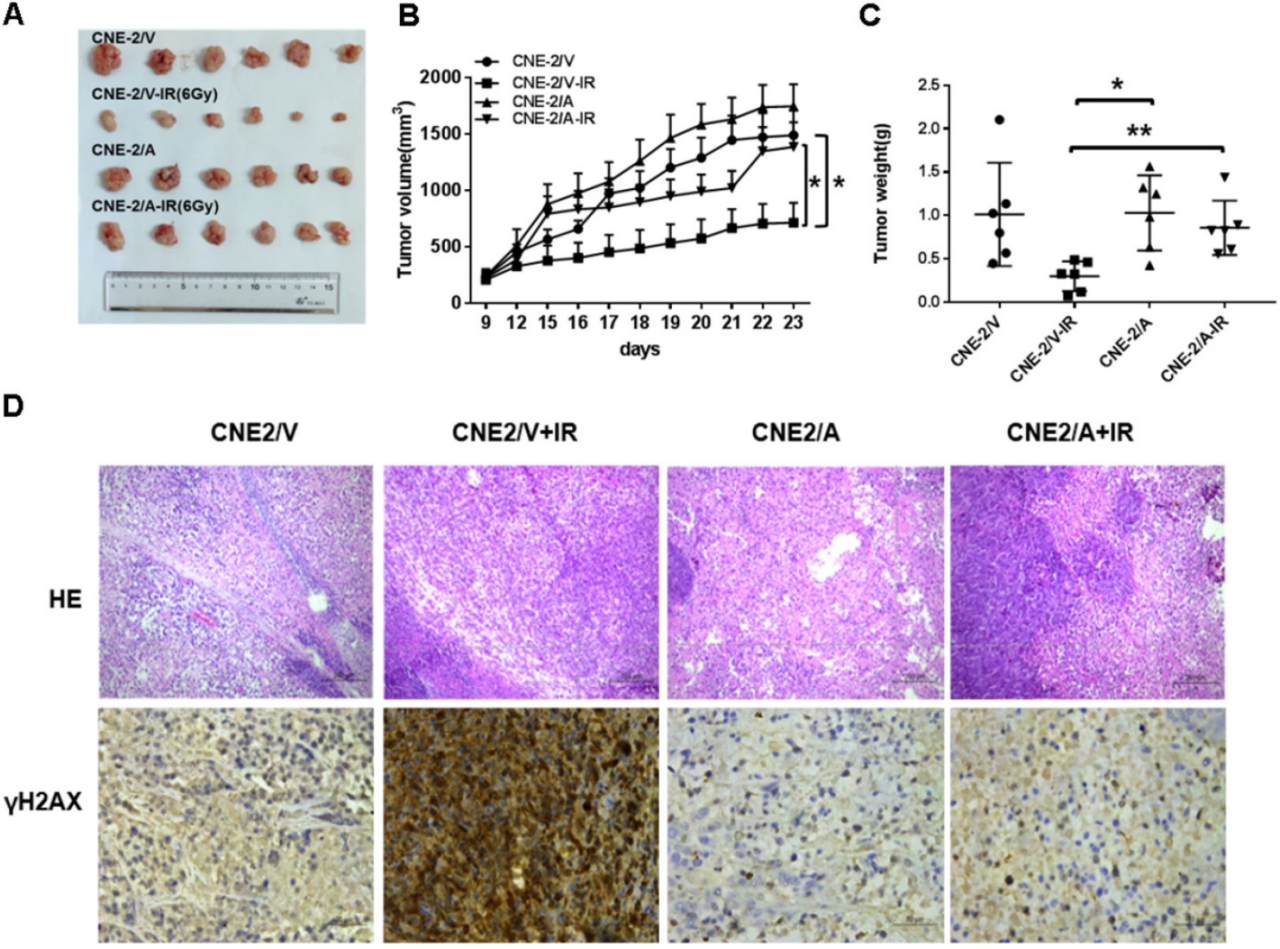
** AKR1B10 promotes NPC radioresistance *in vivo*.** (A) Whole tumors excised from mice injected with CNE-2/Vector, CNE2/Vector+IR, CNE2/AKR1B10, CNE2/AKR1B10+IR. (B and C) Tumor volumes and weight were determined. (D) Analysis of tumor tissue by HE staining and immunohistochemical staining. IR: irradiation; CNE-2/A: CNE-2/AKR1B10, AKR1B10-expressed CNE-2 cells; CNE-2/V: CNE-2/AKR1B10 vector, CNE-2 control cells;*P < 0.05; **P < 0.01.

To evaluate the radioresistence induced by AKR1B10 in an *in vivo* animal model, we established a NPC xenograft mice model by subcutaneously implanted NPC cells (2×10^6^ cells) into balb/c nude mice. When tumor volume reached approximately 100-200 mm^3^, the mice were subjected to IR. However, tumor volume in the experimental group was larger than that in the control group after exposure to 6 Gy IR (Fig. 6A). IR decreased the rate of tumor growth in both the normal control and experimental groups (Fig. 6B). The rate of tumor growth was higher in the CNE-2/AKR1B10 group than that in the CNE-2/Vector group (Fig. 6C). Immunohistologic evaluation of tumor tissues showed that the γH2AX expression level was lower in the CNE-2/AKR1B10 group than that in CNE-2/Vector group (Fig. 6D), suggesting that AKR1B10 promoted radioresistance of NPC *in vivo*.

**Figure 7:**
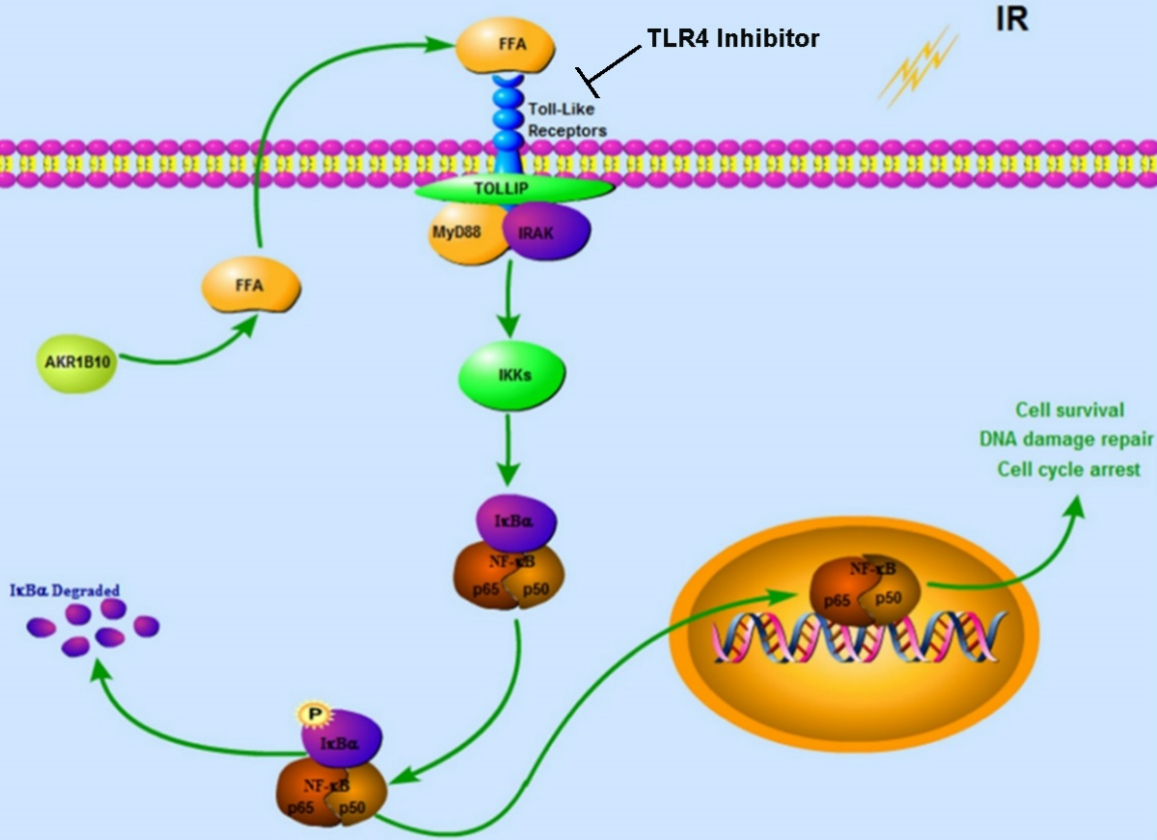
** Proposed Schematic for AKR1B10 activating TLR4/NF-κB signaling pathway by promoting FFA synthesis in NPC.** AKR1B10-induced FFA synthesis activates the TLR4/ NF-κB signaling axis, IKBα phosphorylation and NF-κB nuclear translocation, which then regulates cell cycle arrest and DNA damage repair. IR: irradiation; FFA: Free fatty acid; →, activation or upregulation; ┴ inhibition or downregulation.

Together our data demonstrates that AKR1B10 facilitates radioresistance of NPC via promoting the FFA synthesis and activating TLR4/NF-κB signaling pathway (Fig. 7).

## Discussion

Radiotherapy is the primary mode of treatment used for NPC and the 5-year survival rate of NPC patients in stage I-IVB is about 85 % 28. The main obstacles in the treatment of NPC is the intrinsic and therapy-induced radioresistant behavior of tumor cells. IR can lead to DNA single or double-stranded breaks. Previous research has demonstrated that AKR1B10, which regulates the balance of retinoic acid and lipid metabolism 27, formation of adducts of carbonyl compounds with DNA, blocks DNA replication and RNA transcription, leading to DNA breaks and damage. Thus AKR1B10 promotes cell survival through cytotoxic carbonyl detoxification. In our study, the analysis of resected specimens from NPC radioresistant patients showed that the expression of AKR1B10 is significantly higher compared with radiosensitive patients. Moreover, we also demonstrated that NF-κB activation might be induced by IR *in vitro* and *in vivo*, and NPC cells with constitutive AKR1B10 expression are associated with severe DNA damage outcome after IR. Therefore, we considered that AKR1B10 is involved in therapeutic resistance to radiotherapy in NPC.

AKR1B10 has been shown to be feasible biomarker for radioresistance, infrared IR-induced cell apoptosis and senescence. To study how AKR1B10 affects apoptosis during radiotherapy, we studied the activation of some apoptosis related proteins. The caspase family are important parts in the apoptotic pathway. Caspase-3 is a key executor of apoptosis, which is responsible for the cleavage of many key proteins, such as ribozyme poly (ADP-ribose) polymerase (PARP) 29. Our study suggests that enhanced AKR1B10 level after radiotherapy can inhibit the apoptosis cascade. The main mechanisms by which radiotherapy works are by inducing DNA strand breaks and cell apoptosis.

The repair pathways in place to combat DNA damage include non-homologous end terminal attachment repair and homologous recombination (HR) repair. CHK1 is a responder protein kinase, which can regulate S-phase progression and G2/M cell cycle arrest. When increased, CHK1 upregulation gives more time to repair damaged DNA. Our results shows that AKR1B10 overexpression in NPC cells resulted in increased Chk1 activation phosphorylation after 4 h of IR, stronger DNA repair ability, decreased tumor cell apoptosis, and enhanced radioresistance. However, after 24 h of IR, we found a decrease in chk1 expression, indicating that DNA damage has been repaired. Therefore, AKR1B10 may play a major role in the IR resistance by enhancing DNA repair ability of radioresistant tumor cells.

NF-κB family of transcription factors plays an important role in inflammation and immune response 30, 31. When activated, phosphorylation of the IκB protein and its' rapid degradation through the ubiquitin-proteasome pathway, releases NF-κB into the nucleus and regulates gene expression 32-34. Upon TLR activation, MYD88 recruits IRAK1 (interleukin 1 receptor associated kinase 1) and IRAK4 (interleukin 1 receptor associated kinase 4), forming the NF-κB activation complex and inducing genes encoding inflammatory cytokines expression 35. FFA stimulates the TLR4/NF-κB signaling pathway in adipose and macrophages and is involved in inflammation and tumor progression 36. Treatment with FFA markedly induced the phosphorylation of NF-κB and TLR4 in CNE-2 cells, and knockdown of AKR1B10 in NPC cells resulted in the decrease of NF-κB phosphorylation and TLR4 levels.

In this study, we reported the effect of AKR1B10 on radioresistance of NPCs, and explored the molecular mechanism by which AKR1B10 contributes to this radioresistance. Taken together, we identified a mechanism underlying the association between NF-κB and radioresistance (Fig. 7). AKR1B10-induced FFA synthesis activates the TLR4/NF-κB signaling axis, IKBα phosphorylation and NFκB nuclear translocation which then regulates cell cycle arrest and DNA damage repair. Understanding the mechanism behind radioresistance of NPCs can lead to improved therapeutic approaches, possibly by combination of chemotherapy and radiation.

## Figures and Tables

**Table 1:** The relationship between AKR1B10 expression and the clinical characteristics of the NPC patients
